# The prognostic role of tricuspid annular plane systolic excursion in critically ill patients with septic shock

**DOI:** 10.1186/s44158-025-00227-0

**Published:** 2025-04-30

**Authors:** Tamer Habib, Islam Ahmed, Rasha Abayazeed, Mina Montasser

**Affiliations:** 1 Critical Care Medicine Department, Faculty of Medicine, Alexandria University, Alexandria, 21111 Egypt; 2 Public Health and Community Medicine Department, Faculty of Medicine, Suez-Canal University, Ismailia, Egypt; 3 Pharmacy Practice and Clinical Pharmacy Department, Faculty of Pharmacy, King Salman International University, South-Sinai, El Tor, Egypt; 4 Cardiology and Angiology Department, Faculty of Medicine, Alexandria University, Alexandria, Egypt; 5 Emergency Medicine Department, Faculty of Medicine, Alexandria University, Alexandria, Egypt

**Keywords:** Critical, Septic Shock, TAPSE, Mortality

## Abstract

**Introduction:**

The right ventricle (RV) may play a crucial role in predicting prognosis in critical settings. The value of the tricuspid annular plane systolic excursion (TAPSE) has been shown in the prognosis of cardiac patients, such as those with heart failure and pulmonary hypertension. The aim of this study was to evaluate the possible prognostic performance of RV dysfunction, as assessed by the TAPSE, in noncardiac septic shock patients.

**Methodology:**

One hundred critically ill adult patients diagnosed with septic shock were enrolled directly after admission. The TAPSE was measured within 24 h. Patients were analyzed according to 28-day mortality and divided into non-survivors and survivors.

**Results:**

The overall 28-day mortality rate was 62%. TAPSE showed a strong negative correlation with APACHE-II (*r* = − 0.569, *p* < 0.001) and moderately negatively correlated with the SOFA score (*r* = − 0.448, *p* = 0.001). TAPSE (at a cutoff point of 2 cm) was a very good tool (area under curve = 0.887) for predicting 28-day mortality (95% confidence interval CI 0.770–0.980, *p* < 0.0001).

**Conclusion:**

Early echocardiographic assessment of RV dysfunction to measure TAPSE might be of prognostic importance in noncardiac patients with septic shock, as a TAPSE less than 2 cm was useful for predicting poor outcomes.

**Trial registration:**

clinicaltrials.gov, NCT06008067. Registered 18 July 2023 registered. TAPSESEPTIC study.

## Introduction

Septic shock is defined as “a subset of sepsis in which underlying circulatory and cellular/metabolic abnormalities are profound enough to increase mortality” [1, 2]. Clinically, septic shock can be identified as “the presence of sepsis and persisting hypotension needing vasopressors to maintain mean arterial pressure (MAP) ≥ 65 mmHg and high serum lactate ≥ 2 mmol/L although adequate volume resuscitation”. Septic shock is known to be associated with more than 40% in-hospital mortality [1, 2]. Scoring systems are commonly employed for categorizing septic shock patients. Nonetheless, stratification based solely on categories lacks precision in comparison to a comprehensive scoring system or clinical examination [3, 4].

Transient left ventricular (LV) dysfunction or myocardial stunning has been defined in intensive care unit (ICU) patients as initial LVEF < 40 in non-cardiac patients followed by progressive improvement in the LVEF and segmental contractility until complete normalization had been achieved. In addition to infection or metabolic abnormalities, arrhythmia or stress cardiomyopathy can also occur. Greater numbers of ICU patients with cardiac dysfunction have LV dysfunction according to simple imaging and bedside echocardiography [5–7]. In sepsis, transient LV dysfunction is common and often resolves within 7–10 days as the systemic inflammatory response subsides and hemodynamic stability is restored [8].

The significance of the right ventricle (RV) has recently increased in ICU settings. However, RV function can be difficult to detect and measure. The tricuspid annular plane systolic excursion (TAPSE) is a straightforward and repeatable metric that can be used in a variety of clinical scenarios to evaluate RV function [9, 10].

RV dysfunction in septic shock patients results from a complex interplay of direct myocardial injury, increased afterload (e.g., pulmonary hypertension, mechanical ventilation), systemic hemodynamic alterations, and the RV’s inherent anatomical and physiological vulnerabilities [11–13].

Increased RV afterload in septic shock patients is multifactorial, involving pulmonary hypertension (due to acute respiratory distress syndrome, hypoxic vasoconstriction, or mechanical ventilation), inflammatory mediators, thromboembolic events, ventricular interdependence, and fluid overload [14–16].

It remains uncertain whether RV dysfunction may serve as an indication of severity and is correlated with unfavorable outcomes or heightened morbidity. The RV’s inability to tolerate sudden increases in afterload is due to its anatomical and physiological limitations, increased peripheral vascular resistance from pulmonary hypertension and mechanical ventilation, ventricular interdependence, reduced coronary perfusion, and the effects of fluid overload. These factors collectively make the RV more prone to failure in septic shock [17, 18]. The documented frequency of RV impairment in sepsis patients varies between 31 and 83% [14, 15]. Similar to other factors derived from echocardiographic evaluation, investigations of RV parameters have yielded contrasting findings [19, 20].

The prognostic value of the TAPSE has been shown in cardiac patients, such as those with heart failure and pulmonary hypertension [21–23]. The aim of this study was to evaluate the prognostic performance of RV dysfunction, as assessed by the TAPSE, in noncardiac patients with septic shock.

## Methods

**Fig. 1:**
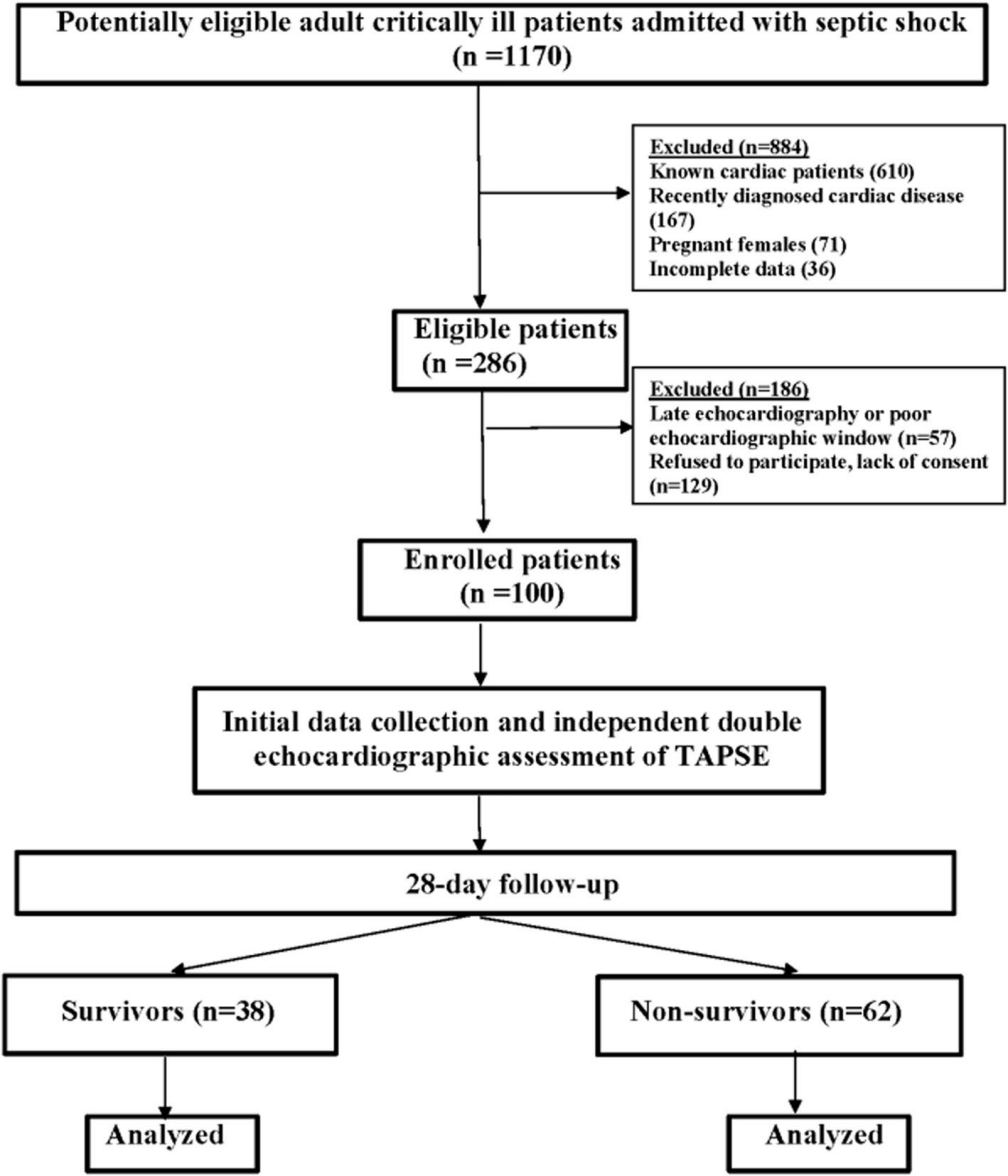
STROBE “Strengthening the Reporting of Observational Studies in Epidemiology” flow chart of the study

This study received ethical approval from the institutional review board of the Faculty of Medicine, Alexandria University (IRB number: 00007589). The study protocol was registered on ClinicalTrials.gov (NCT06008067), and the TAPSESEPTIC cohort study was initiated. All patients who were admitted to the critical care units at Alexandria University Hospitals (AMUH) with Septic Shock (*n* = 1170) over 4 months (April 2023–July 2023) were assessed for enrollment in this study (Fig. 1). Patients were enrolled after formal written informed consent was obtained in a private room from their legal next of kin or guardian. All adult (18–64 years old) patients with the diagnosis of septic shock according to the 2016 consensus definition (Sepsis-3), were enrolled directly after the admission. Exclusion criteria are listed in Table 1.

**Table 1:** The exclusion criteria for TAPSESEPTIC study

Exclusion criterion
Pregnant females
Trauma patients
Documented coronary heart disease
History of myocardial infarction
Myocarditis
Thromboembolic pulmonary disease
Valvular heart disease
Corpulmonale
Arrhythmia
Known LVEF < 40%
Poor echocardiographic window
Lack of informed consent
Patients with incomplete data
Late echocardiographic assessment (after 24 h from admission)

The following admission data were collected from all enrolled patients: complete history, physical examination, acute physiological and chronic health evaluation (APACHE II) score, sequential organ failure assessment (SOFA) score, laboratory investigations and complete sepsis workup. Transthoracic echocardiography was performed using a GE vivid S5 within 24 h after admission. TAPSE was measured in “the apical 4-chamber view as the excursion, in systole, of the junction between the tricuspid annulus and RV free wall by applying the M Mode cursor to this particular point and measuring its longitudinal excursion with RV systole (average of three beats)”. The echocardiographic assessment was revised within the same time frame (24 h) with another formal assessment by a blinded cardiologist to minimize bias.

All patients received standard management for sepsis according to the SCC (sepsis-3) guidelines and the 1-h sepsis bundle [3, 24]. The management protocol was fixed during the study period. The primary endpoint for this study was 28-day mortality, measured as all-cause mortality.

### Statistical analysis

The data was entered into a computer (Microsoft Excel) and analyzed using SPSS 24 (IBM Corp., Armonk, NY, USA). Qualitative data were described using “numbers and percentages”. Quantitative data were described using “the mean and standard deviation, or median”. The significance of the obtained tests was assumed at the 5% level. The receiver operating characteristic (ROC) curve was generated by plotting the sensitivity on the *Y*-axis versus the specificity on the *X*-axis at different cutoff values. The area under the ROC curve denotes “the diagnostic performance of the test”. The Youden index was used to “find the optimal cutoff point to maximize specificity + sensitivity − 1”.

## Results

In this study, one hundred adult patients of both sexes were enrolled and included in the final analysis. The overall 28-day mortality rate was 62%. According to 28-day mortality (the primary endpoint), patients were divided into two groups: survivors and non-survivors. Seventy percent of the overall cohort were males. The median age of all enrolled patients was 55.9 ± 11.1 years. The median SOFA score was 7.2 ± 2.5. The median APACHE II score was 13.4 ± 6.9. Both SOFA and APACHE II scores were significantly elevated in the non-survivors group. The most prevalent suspected source of sepsis was urinary tract infection (44%). The two groups were nearly comparable in their baseline characteristics (Table 2).

**Table 2:** Baseline characteristics of all enrolled patients

	Overall cohort (*n* = 100)	Survivors (*n* = 38)	Non-survivors (*n* = 62)	*p* value
**Male**	70	63.2	74.2	0.528
**Female**	30	36.8	25.8	
**Age (years)**	55.9 ± 11.1	56.4 ± 9.3	55.6 ± 12.2	0.843
**History**				
* Hypertension*	34	16 (42.1)	18 (29)	0.373
* Diabetes*	48	18 (47.4)	30 (48.4)	1.000
* CVS*	26	8 (21.1)	18 (29)	0.741
* CKD*	14	6 (15.8)	8 (12.9)	1.000
* COPD*	18	2 (5.3)	16 (25.8)	0.127
* Hepatic*	10	6 (15.8)	4 (6.5)	0.355
**SOFA score**	7.2 ± 2.5	4.89 ± 1.9	8.65 ± 1.7	< 0.001*
**APACHE II**	13.4 ± 6.9	6.2 ± 2.7	17.9 ± 4.7	< 0.001*
**Source of sepsis**				
* UTI*	44	20 (52.6)	24 (38.7)	0.389
* Chest infection*	34	12 (31.6)	22 (35.5)	1.000
* CNS infection*	8	2 (5.3)	6 (9.7)	1.000
* Abdomen*	10	6 (15.8)	4 (6.5)	0.355
* Blood stream*	6	-	6 (9.7)	0.279
* SSI*	6	-	6 (9.7)	0.279
**MAP (mmHg)**	53.5 ± 7.9	56.4 ± 5.5	53.5 ± 7.9	0.055
**HR (beats/min)**	106.8 ± 13.3	103.5 ± 10.9	108.9 ± 14.3	0.242
**Temp. (ºC)**	37.99 ± 0.9	37.98 ± 0.8	38.00 ± 0.9	0.848
**RR (breath/min)**	31.44 ± 2.5	31.37 ± 2.5	31.48 ± 2.4	0.196
**WBCs × 10**^**9**^**/L**	17.7 ± 11.1	18.6 ± 11.8	17.7 ± 11.1	0.689
**Lactate (mmol/L)**	4.3 ± 2.9	1.9 ± 0.8	5.8 ± 2.7	< 0.001*
**Albumin (g/dL)**	3.0 ± 0.5	2.9 ± 0.4	3.1 ± 0.5	0.060
**CRP (mg/L)**	144.3 ± 75.8	153.3 ± 80.4	138.8 ± 73.7	0.529
**Urea (mg/dL)**	113.3 ± 88.9	92.1 ± 60.7	126.3 ± 101.3	0.254
**S.Cr (mg/dL)**	2.1 ± 1.8	1.6 ± 0.6	2.4 ± 2.2	0.711
**24 h-UOP (mL)**	548.6 ± 225.2	621.6 ± 184.8	503.9 ± 238.5	0.157
**GCS**	12.0 ± 2.6	12.26 ± 2.1	11.84 ± 2.9	0.847
**Troponin-I (ng/mL)**	0.07 (0.002)	0.07 (0.002)	0.08 (0.002)	**0.561**
**BNP (pg/mL)**	512 (75.5)	466 (61.2)	555 (84.3)	0.201
**PASP (mmHg)**	20.82 ± 2.2	20.84 ± **1**.922	20.81 ± 2.400	0.957
**LVEF%**	55.6 ± 7.9	58.0 ± 6.4	54.0 ± 8.6	0.104
**TAPSE (cm)**	1.5 (0.7)	2.1 (0.3)	1.5 (0.2)	< 0.001*

*CVS* cerebrovascular stroke, *COPD* chronic obstructive pulmonary disease, *CKD* chronic kidney disease, *SOFA* sequential organ failure assessment score, *APACHE II* acute physiological and chronic health evaluation-ΙΙ score, *UTI* urinary tract infection, *SSI* skin and soft tissue infections, *MAP* mean arterial pressure, *HR* heart rate, *RR* respiratory rate, *Temp* surface body temperature, *S.Cr* serum creatinine, *UOP* urine output, *GCS* Glasgow coma scale, *BNP *brainnatriureticpeptide,* PASP* pulmonary artery systolic pressure, *LVEF* left ventricular ejection fraction, *TAPSE* tricuspid annular plane systolic excursion. Quantitative data represented as mean ± standard deviation or median (interquartile range). Qualitative data represented as number (%), Fisher exact correction for chi-square test, *p value is significant when *p* ≤ 0.05

**Fig. 2:**
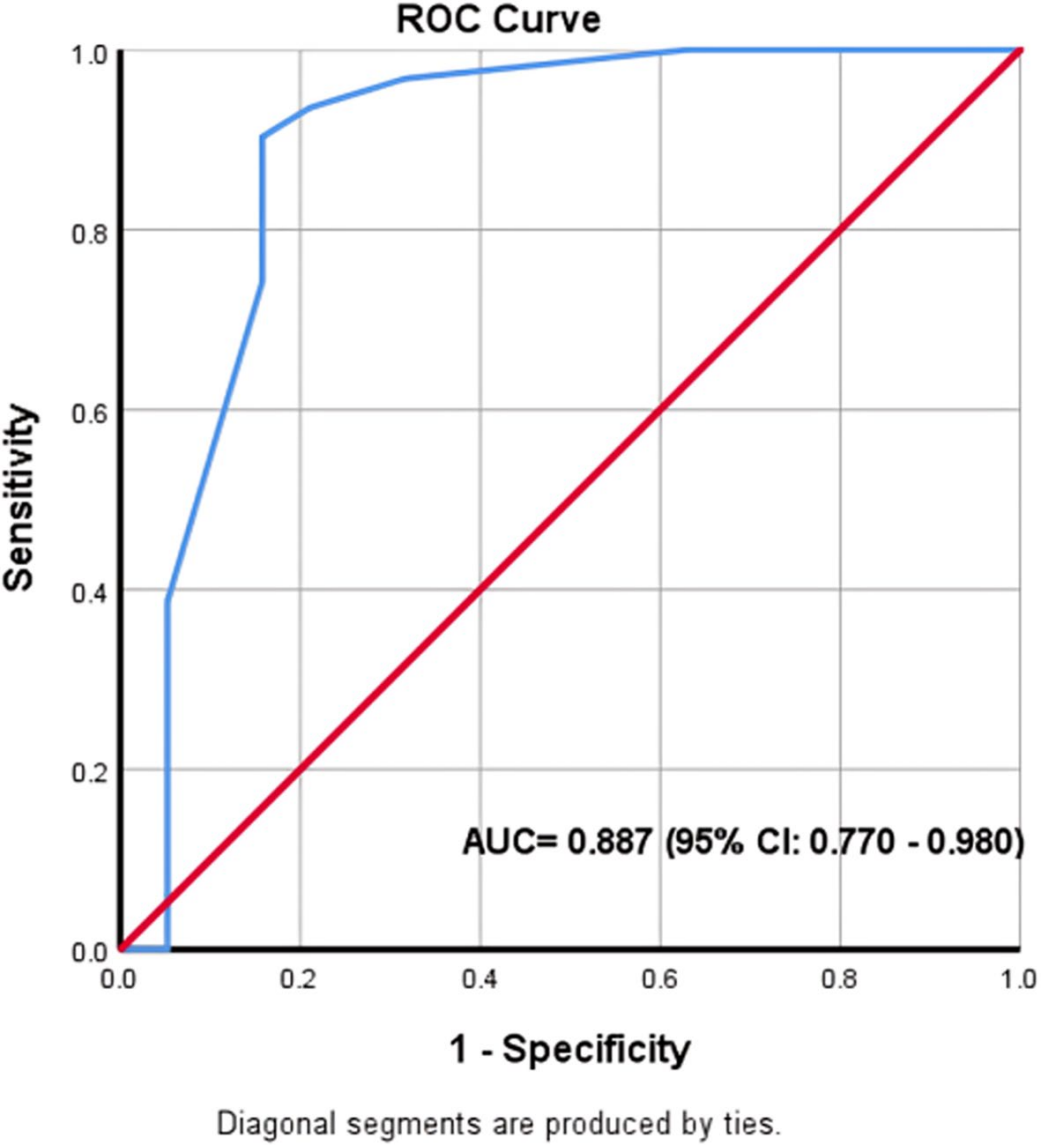
Receiver operating characteristic curve of the sensitivity and specificity of the TAPSE for 28-day mortality (using the Youden index). AUC, area under the curve

The results showed that the median TAPSE of the overall cohort was 1.5 cm. The non-survivors had a significantly lower median TAPSE (1.5 cm) than did the survivors (2.1 cm) (*p* < 0.001). Most patients with low TAPSE were presented with pneumonia and urinary tract infections. TAPSE showed a strong negative correlation with APACHE-II (Spearman’s rho = − 0.569, *p* < 0.001). Similarly, TAPSE is moderately negatively correlated with the SOFA score (Spearman’s rho = − 0.448, *p* = 0.001). According to the ROC curve for the prediction of 28-day mortality in all patients, TAPSE was a very good tool (AUC = 0.887) for the prediction of 28-day mortality (95% CI 0.770–0.980, *p* < 0.0001). It showed good sensitivity (78.95%, 95% CI 54.43% to 93.95%) and excellent specificity (93.55%, 95% CI 78.58% to 99.21%). The positive predictive value was 95.23% (95% CI 83.67% to 98.73%), and the negative predictive value was 73.14% (95% CI 53.15% to 86.73%). The optimal cutoff value was 2 cm for 28-day mortality (Youden index = 0.651) (Fig. 2).

## Discussion

RV function is measured indirectly through the TAPSE. Unlike the LV, which contracts symmetrically in both its transverse and longitudinal axes, the RV contracts largely along its longitudinal axis. Importantly, the septum, which anatomically belongs to the LV, constitutes approximately 40% of the RV. As a marker of RV function, the tricuspid annulus excursion has been utilized for several reasons, including its reliable visibility even in situations with limited RV vision or poor acoustic echo window, as well as the excellent temporal resolution of M-mode acquired data [25].

In this study, most patients with low TAPSE were presented with pneumonia and urinary tract infections. Pulmonary sources of sepsis (e.g., pneumonia) may have a more direct impact on RV function due to increased pulmonary vascular resistance, while non-pulmonary sources may indirectly affect TAPSE through systemic inflammation, fluid resuscitation, or complications like ARDS [26, 27].

In this study, TAPSE showed a strong negative correlation with APACHE-II (*r* = − 0.569, *p* < 0.001) and moderately negatively correlated with the SOFA score (*r* = − 0.448, *p* = 0.001), further supporting the association between impaired RV performance and worsening organ dysfunction. TAPSE less than 2 cm was useful for predicting poor outcomes in noncardiac patients with septic shock in terms of 28-day mortality.

It has been reported that the TAPSE is a reliable indicator of mortality under cardiac conditions. In circumstances involving RV pressure and/or volume overloads, such as heart failure, pulmonary hypertension, and pulmonary embolism [25]. According to the American Society of Echocardiography, an abnormal TAPSE was defined as a TAPSE less than 1.6 cm [28].

In Dong et al.’s [24] retrospective study, the TAPSE was found to be a significant and moderate predictor of both in-ICU mortality (area under the curve (AUC) = 0.762, 95% CI = 0.652–0.871) and 90-day mortality (AUC = 0.69, 95% CI = 0.565–0.814). For both 90-day mortality (sensitivity 80%, specificity 58%) and in-ICU mortality (sensitivity 69%, specificity 77%), the ideal cutoff for the TAPSE was 2.1 cm.

Zhang et al. [28] studied 45 septic shock patients (cases) and 45 non-sepsis patients (controls). There were no statistically significant differences in the LVEF between the two groups (64.6% vs. 67.2%, *p* = 0.161). The mean TAPSE was significantly lower in septic shock patients (1.9 ± 0.4 cm) than in controls (2.3 ± 0.4 cm) (*p* < 0.001). No mortality data were reported for the septic shock group [29].

Gajanana et al. [29] enrolled 120 patients from a mixed population of critically ill patients (with septic shock) with noncardiac illnesses. Echocardiography was performed within 24 h of admission. Based on the ROC curve analysis, a TAPSE less than 2.4 cm was found to be the optimal cutoff point for predicting both short-term and extended mortality. Multivariate analysis revealed that a TAPSE less than 2.4 cm was a noteworthy indicator of in-hospital mortality (*p* = 0.03) [30].

Innocenti et al. [30] enrolled 252 septic patients (40% were shocked), and the 28-day mortality rate was 26%. Using echocardiography within 24 h of admission, RV systolic dysfunction was defined as a TAPSE < 1.6 cm. Cox survival analysis revealed that RV systolic dysfunction predicted increased 28-day mortality (RR = 2.43, 95% CI 1.47–4.00, *p* = 0.001), independent of shock and in addition to LV systolic dysfunction. In sepsis patients, a low TAPSE (< 1.6 cm) predicts 28-day all-cause mortality, independent of LV systolic dysfunction [31].

Zhang et al. [31] demonstrated an association between the ratio of the TAPSE and pulmonary arterial systolic pressure (PASP) and outcomes in septic shock patients on mechanical ventilation. A TAPSE/PASP ratio at an optimal cutoff value of 0.50 mm/mmHg was independently associated with ICU mortality (hazard ratio = 0.027, 95% CI 0.001–0.530, *p* = 0.017) [32].

Similar to the LV, the RV’s function is influenced by its preload, contractility, and afterload. RV failure and venous congestion may result from any one of these factors being compromised. The lack of systemic vascular resistance caused by septic shock may result in decreased preloading. It is well recognized that acute lung injury increases pulmonary vascular resistance, which increases afterload. The relationship between pulmonary hypertension and RV dysfunction in septic shock has been investigated in research, which shows that RV dysfunction occurs independently of pulmonary vascular pressure and shares features with LV dysfunction [25].

In contrast, Lahham et al. [27] performed a pilot study in the emergency department. Twenty-four patients with severe sepsis and septic shock were enrolled, and TAPSE was measured using point-of-care ultrasound. There was no statistically significant association between the TAPSE and mortality (*p* = 0.14) [33].

Additionally, Vallabhajosyula et al. [16] studied 388 adult patients admitted for more than 7 years for severe sepsis or septic shock who underwent echocardiography within 72 h of admission. Fifty-five percent of patients had RV dysfunction, 47% had isolated RV dysfunction, and 53% had combined RV/LV dysfunction. The results did not reveal an association between the TAPSE and in-hospital or 1-year mortality [34].

Singh et al. [34] studied 88 critically ill patients with septic shock within 24 h of admission using echocardiography. Fifty-two patients were categorized as non-survivors, and another 36 were survivors. There were no statistically significant differences in the TAPSE between survivors (23.56 ± 4.45 mm) and non-survivors (23.10 ± 6.67) (*p* = 0.47) [35].

The sensitivity of the TAPSE was found to be significantly high in critical care patients. However, its specificity was lower [36]. This phenomenon could be attributed to the concept of ventricular interdependence. Additionally, the lack of control of acute RV afterload could also have a notable impact on the biventricular relationship. Furthermore, it is worth noting that concomitant improvements in RV/LV ejection fractions may also play a role in elucidating this observation [37, 38]. A prior investigation demonstrated that a significant proportion of RV contraction force, amounting to 30%, emanates from the LV. Hence, during the occurrence of septic shock impacting the LV, the RV is equally affected [39].

This study has several limitations, such as the small sample size for a mortality study in septic shock patients, and lack of prior power calculation. The study design is monocentric. The TAPSE was the only parameter of RV systolic function measured. TAPSE primarily measures longitudinal contraction and may not reflect global RV function. The assessment of RV function in septic shock patients using traditional parameters like TAPSE and fractional area change is increasingly viewed as outdated due to lack of standardization, and inability to capture the complexity of RV dysfunction in this population. Advanced techniques like strain imaging and a focus on RV-pulmonary artery coupling offer more comprehensive insights but are not yet widely adopted. Despite being a parameter that only evaluates longitudinal shortening of the myocardium and angle dependency, the TAPSE is an easy and reproducible measure of RV function that can be rapidly attained in critically ill patients with very good accuracy, so it is a suitable method for use in critical settings. Despite the exclusion criteria, some types of LV dysfunction cannot be ruled out. If TAPSE assessments were conducted at additional points in time, the outcomes would exhibit greater robustness and clinical significance. The catecholamine and acidosis levels were not measured, and the administration of vasopressors was inaccurately documented. The rationale behind mechanical ventilation interplays was not warranted. Consequently, the potential for TAPSE reduction related to ventilator employment cannot be wholly dismissed.

## Conclusions

In light of the available findings, early echocardiographic assessment of RV dysfunction to measure TAPSE might be of prognostic importance in noncardiac patients with septic shock, as a TAPSE less than 2 cm was useful for predicting poor outcomes. Our recommendations are for further larger multicenter studies to find an exact cutoff value for such patients. All vasopressor and mechanical ventilator parameters and interactions should be considered. Echocardiographic assessment of the RV might aid in risk stratification. This may help in the identification of septic shock patients requiring more intensive therapy or interventions based on their RV performance. Finally, the association between RV dysfunction and mortality might offer a new therapeutic target.

## Data Availability

The data is available upon reasonable request. Contact the corresponding author.
